# Downregulation of cathepsin C alleviates endothelial cell dysfunction by suppressing p38 MAPK/NF-κB pathway in preeclampsia

**DOI:** 10.1080/21655979.2021.2023994

**Published:** 2022-01-17

**Authors:** Fan Lu, Han Gong, Houkang Lei, Juan Li

**Affiliations:** a Department of Obstetrics, The Affiliated Hospital of Guizhou Medical University, Guiyang, Guizhou Province, China; b Department of Obstetrics, The Third People’s Hospital of Yunnan Province, Kunming, Yunnan, Province, China

**Keywords:** Cathepsin C, endothelial cell dysfunction, HUVECs, p38 MAPK/NF-κB pathway, preeclampsia

## Abstract

Endothelial cell dysfunction is an essential pathophysiological feature of preeclampsia (PE). It has been reported that cathepsin C is upregulated in the maternal vascular endothelium of PE patients. The excessive activation of p38 MAPK leads to various diseases, including PE. NF-κB pathway can promote uteroplacental dysfunction, endothelial stress and development of PE. Moreover, it has been verified that cathepsin C can activate p38 MAPK/NF-κB pathway. In the present work, hypoxia/reoxygenation (H/R) injury model of HUVECs was established to discuss the biological functions of cathepsin C in endothelial cell dysfunction and to elucidate the underlying molecular mechanism. The correlation between cathepsin C and p38 MAPK/NF-κB pathway in H/R-stimulated HUVECs as well as the effects of cathepsin C and p38 MAPK/NF-κB pathway on viability, apoptosis, invasion, in vitro angiogenesis of HUVECs and oxidative stress were assessed. The results revealed that H/R injury elevated cathepsin C expression and activated p38 MAPK/NF-κB pathway in HUVECs and cathepsin C knockdown inhibited the activity of p38 MAPK/NF-κB pathway in H/R-stimulated HUVECs. Downregulation of cathepsin C improved viability, inhibited apoptosis and enhanced invasion of H/R-stimulated HUVECs. In addition, downregulation of cathepsin C alleviated oxidative stress and induced stronger HUVEC angiogenesis in vitro. Furthermore, the protective effects of cathepsin C knockdown against endothelial cell dysfunction were reversed by p38 MAPK activator anisomycin. In other words, downregulation of cathepsin C could improve HUVEC viability and enhance anti-apoptotic capacity, anti-oxidative capability, invasive ability, as well as angiogenic potential of H/R-stimulated HUVECs by repressing p38 MAPK/NF-κB pathway.

## Introduction

Preeclampsia (PE) is a multisystem progressive disease unique to pregnancy [1]. It is characterized by new-onset of hypertension and proteinuria after 20 weeks of gestation, or new-onset of hypertension and terminal organ dysfunction accompanied with or without proteinuria [2,3]. PE seriously threatens the life safety of pregnant women and fetuses and is closely associated with maternal and fetal mortality worldwide [4]. Oxidative stress at the maternal–fetal interface, inflammation in maternal circulation and disturbance of angiogenesis are the main causes of PE [5–7].

Cathepsin C (CatC), also known as dipeptidyl peptidase I (DPPI), is one of the cathepsins with lysosomal exo-cysteine protease activity [8]. Cathepsin C functions as a central coordinator for the activation of many serine proteases in immune cells [9]. Abnormal expression of cathepsin C may weaken the immune ability of organisms and induce infection and severe inflammation [10,11]. Literature reports that cathepsin C is upregulated in the maternal vascular endothelium of PE patients [12]. However, the biological role of cathepsin C and specific mechanism underling the participation of cathepsin C in the progression of PE have not been fully elucidated till now.

p38 mitogen activated protein kinase (p38 MAPK) is a highly conserved member of the serine/threonine MAPK family whose primary function is to transmit extracellular signals to the cells [13]. p38 MAPK can regulate various pathophysiological processes, such as cell proliferation, differentiation, inflammation, oxidative stress and apoptosis [14,15]. The excessive activation or decreased expression of p38 MAPK leads to various diseases, including PE [16]. It has been verified that p38 MAPK signal pathway can regulate oxidative stress and apoptosis of endothelial cells and affect the angiogenic activity of extra villous trophoblast in PE [17–19]. Hence, p38 MAPK is expected to serve as a therapeutic target for PE.

Nuclear factor-kappaB (NF-κB), a common nuclear transcription factor, is widely present in the cytoplasm, and acts as a signaling pathway when cells are stimulated [20]. As a classic inflammation and oxidative stress signal pathway, NF-κB pathway plays an important role in the body’s inflammatory response, immune regulation and apoptosis by regulating chemotactic cytokines, adhesion molecules, gene expressions of growth factors and various enzymes [21–23]. Importantly, it has been widely reported that NF-κB pathway can promote uteroplacental dysfunction, endothelial stress and development of PE [24,25]. Thus, selective inhibition of NF-κB pathway may be an efficient approach for the treatment of PE.

To sum up, the correlation between cathepsin C and p38 MAPK/NF-κB pathway in H/R-stimulated HUVECs as well as the effects of cathepsin C and p38 MAPK/NF-κB pathway on viability, apoptosis, invasion, in vitro angiogenesis of HUVECs and oxidative stress were assessed, aiming to discuss the biological functions of cathepsin C in endothelial cell dysfunction and to elucidate the underlying molecular mechanism.

## Materials and Methods

### Cell culture

Human umbilical vein endothelial cells (HUVECs) were purchased from the American Type Culture Collection (ATCC, VA, USA) and maintained in Dulbecco’s modified Eagle’s medium (DMEM; Gibco, NY, USA) containing 10% fetal bovine serum (FBS; Gibco, NY, USA) and 1% penicillin/streptomycin in a humidified incubator at 37°C with 5% CO_2_.

### Cell transfection

Cathepsin C siRNA and the control siRNA were obtained from GenePharma (Shanghai, China). For in vitro transfection, HUVECs were transfected with si-cathepsin C or si-NC using Lipofectamine 3000 (Invitrogen, CA, USA) for 48 h following the manufacturer’s instructions.

### Cell treatment

To investigate the specific effects of p38 MAPK pathway, HUVECs were pre-incubated with or without p38 MAPK activator anisomycin (25 μg/ml; Cell Signaling Technology, MA, USA) for 30 min.

### Hypoxia/reoxygenation (H/R) injury model of HUVECs

To establish H/R injury model of HUVECs, HUVECs were cultured in preconditioned hypoxic medium under 5% CO_2_ and 95% N2 in a humidified incubator for 8 h and then replaced with a fresh medium. Afterward, HUVECs were cultured under normal growth conditions for 16 h of reoxygenation [26].

### Cell counting kit 8 (CCK-8) assay

The viability of the HUVECs was measured by CCK-8 assay. In short, HUVECs were cultured in 96-well plates (1 × 10^4^ cells/well) for 24 h. Then, CCK-8 solution (Beyotime, Shanghai, China) was added into each well, and incubated for another 4 h. The absorbance was measured at 450 nm with a microplate reader (Bio-Rad, CA, USA).

### Cell apoptosis assay

The proportions of apoptotic cells were determined using FITC Annexin V Apoptosis Detection Kit (BD Bioscience, CA, USA). Briefly, HUVECs (1 × 10^6^) were harvested and then washed twice with ice-cold PBS. Then, HUVECs were resuspended in 400 μl of 1× binding buffer, and cell suspension was incubated with Annexin V and PI in the dark for 15 min at room temperature. Next, HUVECs were assessed by a FACS flow cytometer (BD Biosciences, CA, USA).

### Malondialdehyde (MDA) and superoxide dismutase (SOD) detection

HUVECs were harvested and washed with PBS. Next, HUVECs were incubated with the lysis buffer (Beyotime, Shanghai, China), and cell supernatant of lysates was collected by centrifugation. Levels of MDA and SOD were analyzed using MDA assay kit and SOD assay kit (Jiancheng Biotech, Nanjing, China).

### Transwell invasion assay

HUVECs were resuspended in a serum-free medium and seeded onto the upper chamber of transwell chambers (Millipore, MA, USA) precoated Matrigel (BD Biosciences, CA, USA). Next, FBS-containing DMEM was placed to the lower chamber. After 24 h incubation, the invaded cells were fixed with 4% paraformaldehyde for 30 min and stained with 0.1% crystal violet (Solarbio, Beijing, China) for 30 min. The penetrated cells were photographed and counted under an inverted microscope (Nikon, Tokyo, Japan).

### Tube formation assay

HUVECs were trypsinized, resuspended with a serum-free medium and seeded (2 × 10^4^ cells/well) into 96-well plates precoated with 50 μl Matrigel (BD Biosciences, CA, USA). After 24 h of incubation, tube formation was observed and photographed under an inverted microscope (Nikon, Tokyo, Japan). Quantitative analysis of tubules was performed using AngioSys 2.0 image analysis software (Cellworks, Buckingham, UK).

### Reverse transcription-quantitative polymerase chain reaction (RT-qPCR)

Total RNA was isolated from HUVECs using TRIzol reagent (Invitrogen, CA, USA) following the manufacturer’s instructions. 1 µg RNA was reversely transcribed into cDNA using a reverse transcription kit (Takara, Tokyo, Japan). Next, PCR reactions were performed on ABI 7500 quantitative PCR instrument using SYBR Master Mixture (Takara, Tokyo, Japan). The PCR conditions were as follows: 95°C for 10 min, followed by 40 cycles of 95°C for 15 sec and 60°C for 60 sec. The primers were as following: cathepsin C, forward: 5′- CCAACTGCACCTATCTTGACC −3′ and reverse: 5′- AAGGCAAACCACTTGTAGTCATT −3′; GAPDH, forward: 5′- GAAGGTGAAGGTCGGAGTC −3′ and reverse: 5′- GAAGATGGTGATGGGATTTC −3′. The relative expression of mRNA was normalized to GAPDH and calculated using 2^–ΔΔCt^ method.

### Western blotting analysis

HUVECs were lysed in ice-cold RIPA lysis buffer (Beyotime, Shanghai, China), and protein concentration was measured by BCA Protein Assay Kit (Beyotime, Shanghai, China). Equal amounts of protein samples were separated by sodium dodecyl sulfate-polyacrylamide gel electrophoresis (SDS-PAGE) and transferred to PVDF membranes. Next, membranes were blocked with 5% BSA for 1 h at room temperature and then incubated overnight 4°C with the primary antibodies as follows: cathepsin C (Abcam, ab199109, 1:1000), p-p38 MAPK (Abcam, ab178867, 1:1000), p38 MAPK (Abcam, ab170099, 1:5000), p-NF-κB p65 (Abcam, ab76302, 1:1000), NF-κB p65 (Abcam, ab32536, 1:10,000), p-IκBα (Abcam, ab133462, 1:10,000), IκBα (Abcam, ab32518, 1:10,000), Bcl-2 (Abcam, ab196495, 1:2000), Bax (Abcam, ab53154, 1:1000), Birc5 (Abcam, ab76424, 1:5000), Cleaved PARP (Abcam, ab32064, 1:10,000), PARP (Abcam, ab191217, 1:1000) and GAPDH (Abcam, ab9485, 1:2500). On the second day, the membranes were incubated with the corresponding secondary antibody (Abcam, ab205718, 1:50,000) for 1.5 h at room temperature. Protein blots were visualized using electrochemiluminescence (ECL; Beyotime, Shanghai, China) method and analyzed by a Bio-Rad imaging system (Bio-Rad, CA, USA).

### Statistical analysis

Each experiment is performed in triplicate. Experimental data were analyzed by one-way analysis of variance (ANOVA) followed by Tukey’s post hoc test using GraphPad Prism 7.00 and expressed as mean ± standard deviation. Differences in statistical significance were set at p < 0.05. *p < 0.05, **p < 0.01, ***p < 0.001

## Results

### Enhanced cathepsin C expression and activated p38 MAPK/NF-κB pathway in H/R-stimulated HUVECs

**Figure 1.**
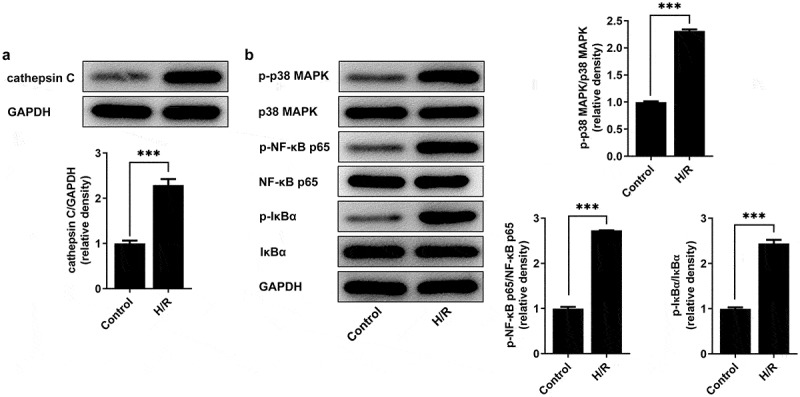
Enhanced cathepsin C expression and activated p38 MAPK/NF-κB pathway in H/R-stimulated HUVECs. (a) Western blot assay was employed to detect cathepsin C expression. (b) Western blot assay was employed to detect expressions of p-p38 MAPK, p38 MAPK, p-NF-κB p65, NF-κB p65, p-IκBα and IκBα. *** p < 0.001.

The biological roles of cathepsin C and p38 MAPK/NF-κB pathway in H/R injury model of HUVECs were evaluated. H/R injury enhanced cathepsin C expression in HUVECs (Figure 1(a)). Besides, elevated expressions of p-p38 MAPK, p-NF-κB p65 and p-IκBα evidenced that p38 MAPK/NF-κB pathway was activated in H/R-stimulated HUVECs (Figure 1(b)).

### Downregulation of cathepsin C inhibited the activity of p38 MAPK/NF-κB pathway in H/R-stimulated HUVECs

**Figure 2.**
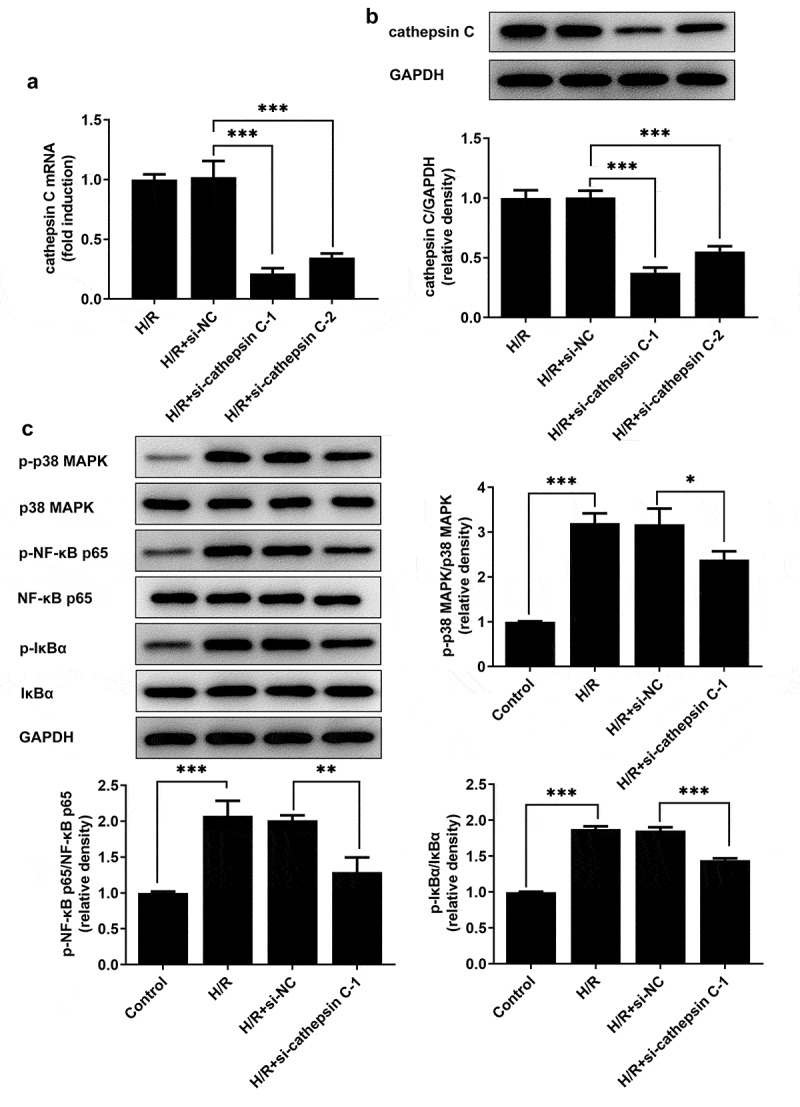
Downregulation of cathepsin C inhibited the activity of p38 MAPK/NF-κB pathway in H/R-stimulated HUVECs. (a) HUVECs were transfected with si-cathepsin C-1 or si-cathepsin C-2. Western blot assay was employed to detect cathepsin C protein expression to validate transfection efficiency. (b) HUVECs were transfected with si-cathepsin C-1 or si-cathepsin C-2. RT-qPCR was employed to detect cathepsin C mRNA level to validate transfection efficiency. (c) Western blot assay was employed to detect expressions of p-p38 MAPK, p38 MAPK, p-NF-κB p65, NF-κB p65, p-IκBα and IκBα. * p < 0.05, ** p < 0.01, *** p < 0.001.

To elucidate the correlation between cathepsin C and p38 MAPK/NF-κB pathway, H/R-stimulated HUVECs were transfected with si-cathepsin C-1 or si-cathepsin C-2, and then the activity of p38 MAPK/NF-κB pathway was assessed. Transfection with si-cathepsin C-1 or si-cathepsin C-2 distinctly downregulated cathepsin C expression (Figure 2(a, b)). Due to the optimized transfection efficiency, si-cathepsin C-1 was selected for subsequent analysis. Decreased expressions of p-p38 MAPK, p-NF-κB p65 and p-IκBα indicated that activation of p38 MAPK/NF-κB pathway in H/R-stimulated HUVECs was inhibited by downregulation of cathepsin C (Figure 2(c)).

### Downregulation of cathepsin C enhanced viability and inhibited apoptosis of H/R-stimulated HUVECs by repressing p38 MAPK/NF-κB pathway

**Figure 3.**
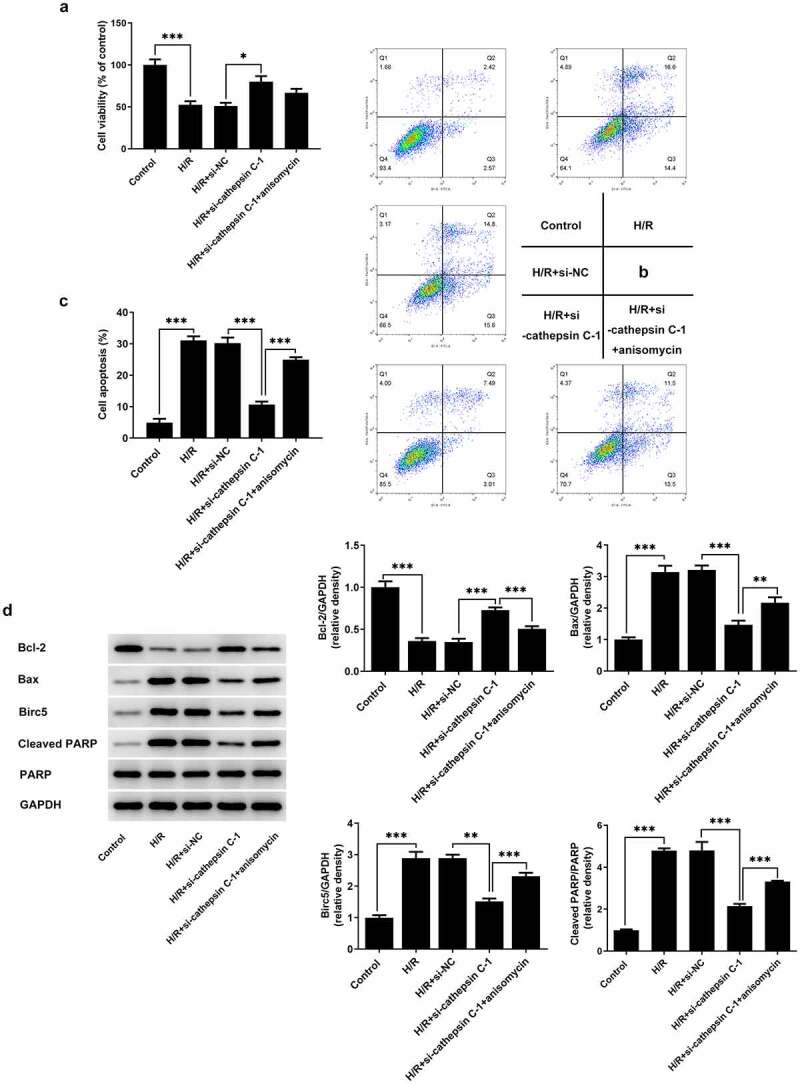
Downregulation of cathepsin C enhanced viability and inhibited apoptosis of H/R-stimulated HUVECs by repressing p38 MAPK/NF-κB pathway. (a) CCK-8 assay was employed to detect HUVEC viability. (b, c) Flow cytometry analysis was employed to detect apoptosis of HUVECs. (d) Western blot assay was employed to detect expressions of Bcl-2, Bax, Birc5, cleaved PARP and PARP. * p < 0.05, ** p < 0.01, *** p < 0.001.

H/R injury led to decreased HUVEC viability and increased apoptosis of HUVECs. Downregulation of cathepsin C improved the viability of H/R-stimulated HUVECs, which was partially abrogated by treatment with p38 MAPK activator anisomycin (Figure 3(a)). Moreover, downregulation of cathepsin C repressed apoptosis of H/R-stimulated HUVECs and anisomycin reversed the suppressing effects of cathepsin C knockdown on apoptosis of H/R-stimulated HUVECs (Figure 3(b, c)). Besides, decreased Bcl-2 expression and increased expressions of Bax, Birc5 and cleaved PARP were observed in H/R-stimulated HUVECs. Downregulation of cathepsin C elevated Bcl-2 expression and reduced expressions of Bax, Birc5 and cleaved PARP, which were reversed upon anisomycin treatment (Figure 3(d)). In other words, cathepsin C knockdown improved viability and suppressed apoptosis of H/R-stimulated HUVECs by repressing p38 MAPK/NF-κB pathway.

### Downregulation of cathepsin C alleviated oxidative stress in H/R-stimulated HUVECs by repressing p38 MAPK/NF-κB pathway

**Figure 4.**
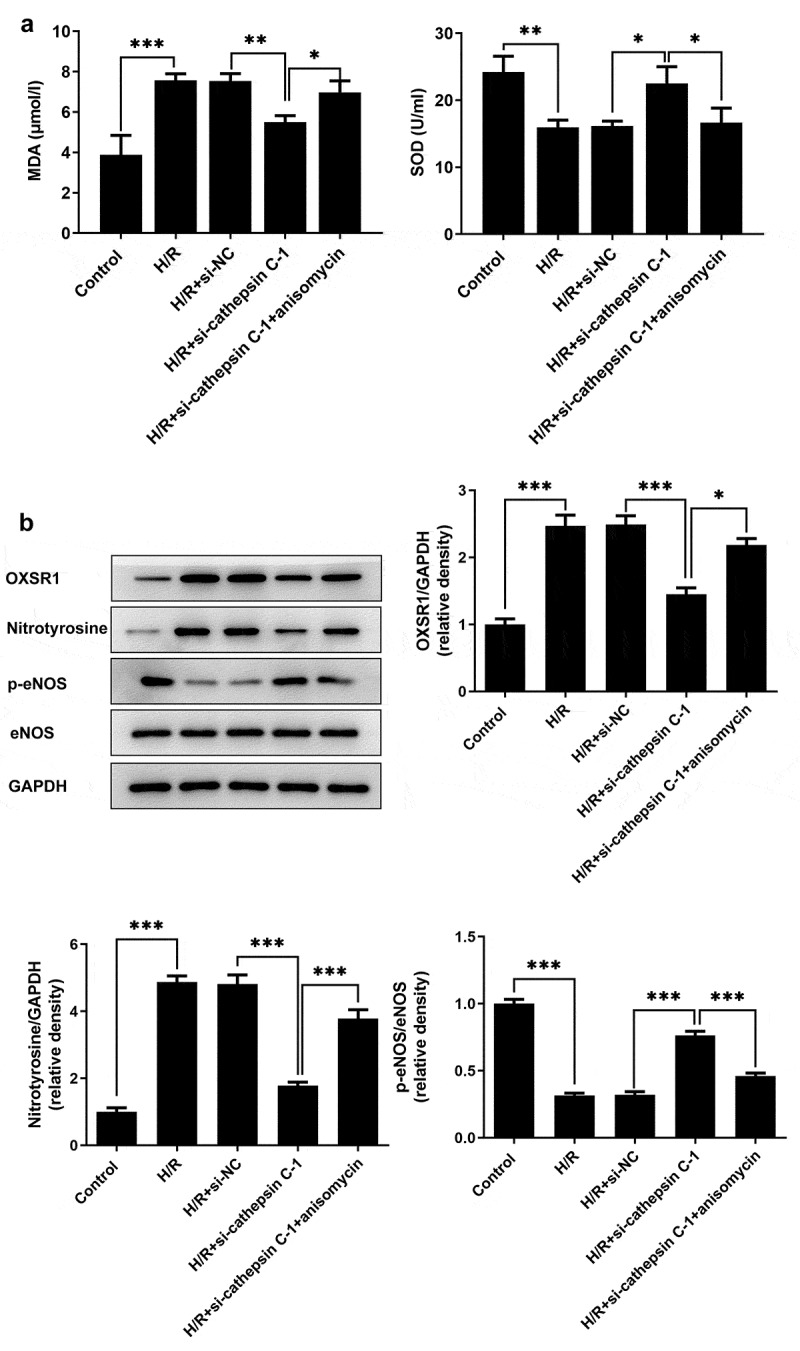
Downregulation of cathepsin C alleviated oxidative stress in H/R-stimulated HUVECs by repressing p38 MAPK/NF-κB pathway. (a) MDA assay kit and SOD assay kit were employed to detect MDA and SOD levels. (b) Western blot assay was employed to detect expressions of OXSR1, Nitrotyrosine, p-eNOS and eNOS. * p < 0.05, ** p < 0.01, *** p < 0.001.

H/R injury increased the levels of MDA, OXSR1 and Nitrotyrosine as well as decreased the levels of SOD and p-eNOS in HUVECs. Downregulation of cathepsin C reduced the levels of MDA, OXSR1 and Nitrotyrosine as well as elevated the levels of SOD and p-eNOS in H/R-stimulated HUVECs. Furthermore, anisomycin treatment reversed the regulating effects of cathepsin C knockdown on the levels of oxidative stress associated markers (Figure 4(a, b)). In summary, cathepsin C knockdown mitigated oxidative stress in H/R-stimulated HUVECs by repressing p38 MAPK/NF-κB pathway.

### Downregulation of cathepsin C boosted invasion of H/R-stimulated HUVECs by repressing p38 MAPK/NF-κB pathway

**Figure 5.**
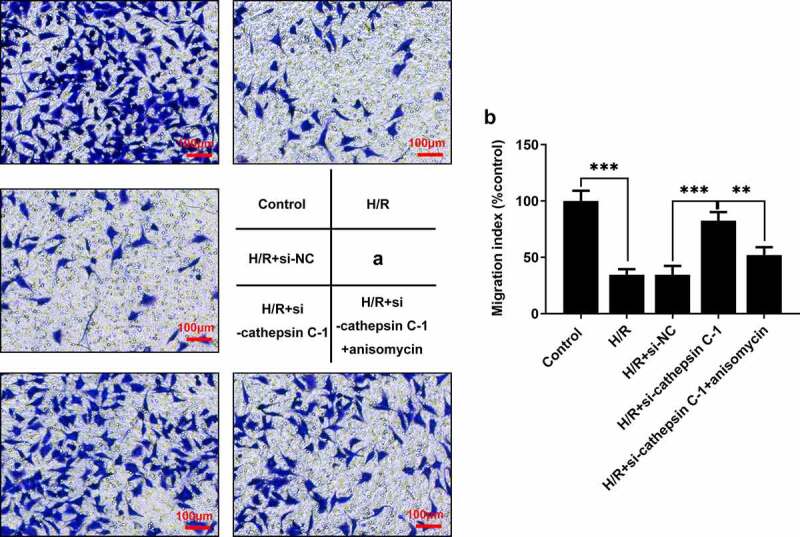
Downregulation of cathepsin C boosted invasion of H/R-stimulated HUVECs by repressing p38 MAPK/NF-κB pathway. (a, b) Transwell assay was employed to detect invasion of HUVECs. ** p < 0.01, *** p < 0.001.

Attenuated invasion of HUVECs was observed in H/R-stimulated HUVECs. Downregulation of cathepsin C enhanced invasion of H/R-stimulated HUVECs and the promoting effect of cathepsin C knockdown on invasion of HUVECs was partly abolished by anisomycin treatment (Figure 5(a, b)).

### Downregulation of cathepsin C induced stronger HUVEC angiogenesis in vitro by repressing p38 MAPK/NF-κB pathway

**Figure 6.**
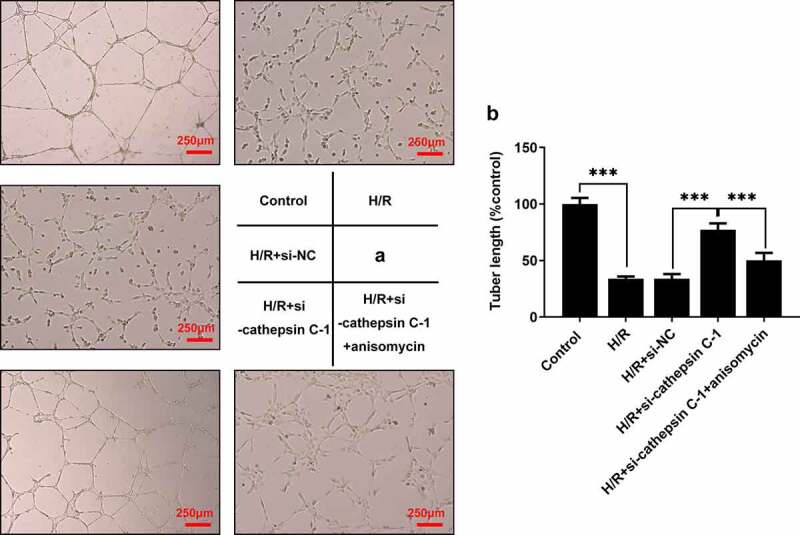
Downregulation of cathepsin C induced stronger HUVEC angiogenesis in vitro by repressing p38 MAPK/NF-κB pathway. (a, b) Tube formation assay was employed to detect HUVEC angiogenesis in vitro. *** p < 0.001.

Results of tube formation assay revealed that H/R injury resulted in a week in vitro angiogenesis in HUVECs. Downregulation of cathepsin C strengthened in vitro angiogenesis in H/R-stimulated HUVECs. The enhancing effect of cathepsin C knockdown on HUVEC angiogenesis in vitro was partially abrogated by anisomycin treatment (Figure 6(a, b)).

## Discussion

It has been demonstrated that the main causes of PE include abnormal remodeling of spiral arteries in placenta, placental defects, oxidative stress at maternal–fetal interface, inflammation in maternal circulation and disturbance of angiogenesis. These events result in a systemic and diffuse endothelial cell dysfunction, an essential pathophysiological feature of PE [6,7].

Cathepsin C is a lysosomal cysteine protease belonging to the papain superfamily [8]. Cathepsin C can activate serine proteases and secrete serine proteases into the microenvironment to produce proinflammatory effects [27]. Nowadays, cathepsin C has received increasing attention due to its biological roles in various systemic diseases, such as tumor, osteoporosis and rheumatoid arthritis [28–30]. However, the specific role of cathepsin C in the progression of PE is rarely discussed. Gu et al. [12] report that cathepsin C is markedly upregulated in the maternal vascular endothelium of PE patients compared with normal pregnant controls. Besides, literature proves that cathepsin C is highly expressed in high glucose-treated HUVECs [31]. Researches above indicate that cathepsin C is closely associated with the occurrence and development of endothelial cell dysfunction. In this investigation, elevated cathepsin C expression was observed in H/R-stimulated HUVECs and H/R injury led to HUVEC dysfunction. Downregulation of cathepsin C improved HUVEC viability and enhanced anti-apoptotic capacity, anti-oxidative capability, invasive ability, as well as angiogenic potential of H/R-stimulated HUVECs, protecting against H/R-induced HUVEC dysfunction.

Previous reports indicate that p38 MAPK/NF-κB signaling pathway plays a vital role in PE progression. Zhao et al. [17] discover that H_2_O_2_ treatment could promote p38 MAPK phosphorylation in HUVECs, while p38 MAPK inhibitor could restrain H_2_O_2-_induced apoptosis and alleviate PE-related endothelial cell injury. Kim et al. [24] report that NF-κB inhibitor or NF-κB p65 knockdown could downregulate miR-31-5p expression in human endothelial cells to suppress endothelial cell dysfunction of PE. Besides, researchers prove that cathepsin C could activate p38 MAPK/NF-κB pathway [32,33]. In this present work, H/R injury activated p38 MAPK/NF-κB pathway in HUVECs and cathepsin C knockdown inhibited the activity of p38 MAPK/NF-κB pathway in H/R-stimulated HUVECs. Cathepsin C knockdown improved viability, inhibited apoptosis and enhanced invasion of H/R-stimulated HUVECs, alleviated oxidative stress as well as induced stronger HUVEC angiogenesis in vitro, which were reversed by anisomycin treatment.

## Conclusion

To conclude, downregulation of cathepsin C improved HUVEC viability and enhanced anti-apoptotic capacity, anti-oxidative capability, invasive ability, as well as angiogenic potential of H/R-stimulated HUVECs by repressing p38 MAPK/NF-κB pathway. The study on the protective mechanism of cathepsin C knockdown against endothelial cell dysfunction may suggest a novel promising approach for PE therapies.

## Data Availability

The analyzed data sets generated during the present study are available from the corresponding authors on reasonable request.
